# Regression calibration for time-to-event outcomes: mitigating bias due to measurement error in real-world endpoints

**DOI:** 10.1515/em-2025-0009

**Published:** 2025-09-26

**Authors:** Benjamin Ackerman, Ryan W. Gan, Youyi Zhang, Juned Siddique, James Roose, Jennifer L. Lund, Janick Weberpals, Jocelyn R. Wang, Craig S. Meyer, Jennifer Hayden, Khaled Sarsour, Ashita S. Batavia

**Affiliations:** 426218 Johnson & Johnson, Raritan, NJ, USA; Preventive Medicine and Psychiatry and Behavioral Science, Feinberg School of Medicine, Northwestern University, Chicago, IL, USA; Flatiron Health, New York, NY, USA; Department of Epidemiology, University of North Carolina at Chapel Hill, Chapel Hill, NC, USA; Division of Pharmacoepidemiology and Pharmacoeconomics, Brigham and Women’s Hospital, Harvard Medical School, Boston, MA, USA

**Keywords:** measurement error, real-world data, real-world evidence, time to event, calibration, endpoints

## Abstract

**Objectives:**

In drug development, there is increasing interest in leveraging real-world data (RWD) to augment trial data and generate evidence about treatment efficacy. However, comparing patient outcomes across trial and routine clinical care settings can be susceptible to bias, namely due to differences in how and when disease assessments occur. This can introduce measurement error in RWD relative to trial standards and lead to bias when comparing endpoints. We develop a novel statistical method, survival regression calibration (SRC), to mitigate measurement error bias in time-to-event RWD outcomes and improve inferences when combining trials with RWD in oncology.

**Methods:**

SRC extends upon existing regression calibration methods to address measurement error in time-to-event RWD outcomes. The method entails fitting separate Weibull regression models using trial-like (‘true’) and real-world-like (‘mismeasured’) outcome measures in a validation sample, and then calibrating parameter estimates in the full study according to the estimated bias in Weibull parameters. We evaluate performance of SRC under varying degrees of existing measurement error bias via simulation, and then illustrate how SRC can address measurement error when estimating median progression-free survival (mPFS) in newly diagnosed multiple myeloma RWD.

**Results:**

When measurement error exists between trial and real-world mPFS, SRC can effectively account for its resulting bias. SRC yields greater reduction in measurement error bias than standard regression calibration methods, due to its suitability for time-to-event outcomes.

**Conclusions:**

Outcome measurement error is important to address when combining trials and RWD, as it may lead to biased results. Our SRC method helps mitigate such bias, improving comparability between real-world and trial endpoints and strengthening evidence of treatment efficacy.

## Introduction

When evaluating the efficacy of new therapies, it is important to collect measurements of the outcome under study that are reliable, accurate, and reflective of the underlying true disease state. In clinical trials, endpoints are constructed from outcomes that are measured per strict protocol definitions, often entailing independent review, confirmation, and/or complete biomarker data collection. Increasingly, research in ‘real-world’ settings is being conducted across the drug development landscape, from discovery and regulatory submission to post-approval and post-marketing. Such research includes prospective observational studies or retrospectively collected electronic health records or administrative healthcare claims, commonly referred to as ‘real-world data’ (RWD) [1]. In RWD, outcome collection may be less regimented or complete when compared to a clinical trial. For instance, in RWD, endpoints may be derived by combining specific data elements abstracted from unstructured clinical notes, lab reports, and/or patient-reported data. Outcomes may also be heterogeneous, and dependent on local or regional variations in clinical practice. Furthermore, missingness in documents (e.g., pathology reports, genomic data, image files) or structured data fields could be present. These factors could all contribute to differences in how and when outcomes are captured between RWD and trials, resulting in measurement error [2]. Our work is motivated by the need to understand and address differences in how outcomes are measured between trial settings and real-world settings, namely when constructing external comparator arms in oncology.

Statistical methods exist to address mismeasured variables, like those that may appear in RWD [3], [4], [5], [6], [7], [8]. Broadly, these methods entail obtaining a ‘validation sample,’ in which both the true and mismeasured variables are collected for a group of patients, and a model is fit to estimate their relationship. Validation samples can either be *internal*, meaning that true variables are collected on a sub-population of the main study of interest, or *external*, meaning that true variables (and mismeasured variables) are collected for a completely separate set of patients from the main study of interest. The models are then used to adjust (or calibrate) mismeasured variables in the full sample of interest. While much of the existing literature has focused on understanding and reducing bias due to misclassified exposure status or mismeasured covariates, less attention has been paid to mismeasured *outcomes* [9], [10], [11]. Nevertheless, outcome measurement error remains a critical concern for estimating treatment effectiveness [12].

Within the literature on outcome measurement error, there is emphasis on outcomes that are normally distributed or binary [13], 14], while methods suitable for time-to-event outcomes are less studied. Time-to-event endpoints, like overall survival (OS), and the composite endpoint of progression-free survival (PFS), are commonly used in oncology studies. Additionally, much of the methodological work to date has focused on estimating causal contrasts between two (or more) groups, reflecting a treatment effect [15], [16], [17], rather than a summary measure for a single group. However, the latter is particularly important when considering single-arm clinical trials that use external controls, in which RWD may be used to construct a ‘standard of care’ benchmark to contextualize the trial results. In this case, outcomes are assumed to be measured *without* error in the single-arm trial, and potentially measured *with error* in the comparator arm, *relative to trial standards*.

Few methods for time-to-event outcome measurement error correction exist, and their relevance and applicability to oncology RWD endpoints is limited. While Giganti et al. (2020) proposed a multiple imputation approach to correct for misclassified event status over time when estimating cumulative incidence of AIDS-defining events among patients with HIV, the method is highly susceptible to model misspecification and reliant on the existence of large validation samples [18]. Edwards et al. (2019) and Bakoyannis and Yiannoutsos (2015) derived a cumulative incidence estimator that accounts for time-varying rates of misclassified false positive and false negative events [19], 20]; however, this approach accounts for misclassified event status, not mismeasured event times. Oh et al. (2021) assumed that error in the time-to-event outcome is additive, and used linear regression to estimate bias between the true and mismeasured outcomes [21], then calibrated the mismeasured outcomes in the full sample accordingly. However, modeling a time-to-event outcome in this way may perform sub-optimally when data are right-censored, as often occurs in the trial and real-world settings of interest. Nevertheless, regression calibration is an appealing and established approach for handling other types of mismeasured variables [7], and further refinement to address time-to-event outcome measurement error is warranted. Accordingly, the aim of this work is to develop regression calibration methods to be more suitable for time-to-event outcome data by reframing measurement error in terms of a Weibull model parameterization.

The remainder of the paper is structured as follows. First, we summarize available regression calibration methods for mitigating outcome measurement error in continuous outcomes and highlight the limitations when applied to time-to-event outcomes. We then introduce refined framing and parameterization of the measurement error problem and extend regression calibration methods accordingly. Through simulation, we evaluate our proposed method and compare its performance to standard regression calibration approaches. Next, using the control arm of a historical trial in newly diagnosed multiple myeloma (NDMM), we demonstrate the application of our method, comparing estimates of median PFS under trial and RWD-like conditions. Lastly, we discuss the advantages and limitations of our method, highlighting the importance of novel methods for mitigating measurement error in real-world time-to-event oncology endpoints.

## Materials and methods

Throughout this paper, we will consider a motivating example in NDMM, with a primary goal of estimating median PFS in a real-world dataset. We will consider that, for each patient, the outcome Y (time to a progression event or death), along with relevant baseline covariates X, are collected in the RWD. Furthermore, we will consider that all RWD patients have the potentially *mismeasured* version of Y collected (i.e., criteria for determining the event may be applied differently and assessments may differ), while a small subset of patients is sampled into an internal validation sample, and therefore also has the *ground truth* Y collected (i.e., these patients are also assessed per randomized trial ‘gold standard’ criteria).

Let Y_i_ be the event times measured without error for patient i, i=1, …, n, and Y_i_* be the event time measured *with* error. A standard way to define outcome measurement error is by assuming an additive error structure:

$$Y_{i}^{*}=Y_{i}+\gamma_{1}+\gamma_{X}X+\varepsilon =Y_{i}+\omega$$

where X is a baseline covariate, *γ*_1_ and *γ*_*X*_ are parameters describing the measurement error magnitude, and *ɛ* is a random variable with mean 0. For simplicity, we will assume a nondifferential error structure such that *ω*=*γ*_1_ (i.e., an ‘intercept-only’ model). With this formulation, the standard regression calibration (RC) approach broadly entails estimating *ω* in a validation sample (a necessary source of data where both Y and Y* are collected), calibrating Y_i_* in the full sample by subtracting the estimate of *E *[*ω*], and using the calibrated outcome to estimate the quantity of interest. However, with time-to-event outcomes where the quantity of interest may be a marginal median failure time (i.e., using a Kaplan–Meier estimator), assuming an additive linear error structure may not be appropriate, and can be viewed as a form of model misspecification. In practice, using an additive linear model to calibrate time-to-event outcome measurement error may lead to negative times for some patients. Consider a simple example where we collect true and mismeasured outcomes for a subset of 10 patients in a broader study (See Table 1). On average, regression calibration would reduce error between true and mismeasured times, but for some patients with shorter observed times, this would yield negative calibrated times, which is not possible to observe. This scenario could occur in practice if measurement error leads to large biases in observed outcomes and if there is heterogeneity in event times among patients, which may be plausible in RWD sources like electronic health records [2]. Furthermore, when event rates are low and censoring rates are high, there may only be a small subset of patients for whom the outcome is measured with error. In such cases, typical regression calibration could entail fitting linear models to zero-inflated data. Lastly, note that while this approach calibrates event *times*, it fails to account for mismeasurement in the event *status*, which could further lead to erroneous survival curve estimation.

**Table 1:** Illustrative regression calibration example, in which negative calibrated event times are produced.

*Y*	*Y* ^*^	*ω*=*Y* ^*^–*Y*	*E* [*ω*]	*Ŷ*=*Y* ^*^–*E* [*ω*]
10	10	0	3.5	6.5
11	15	4		11.5
3	12	9		8.5
2	2	0		−1.5
4	3	−1		−0.5
6	7	1		3.5
3	9	6		5.5
3	5	2		1.5
6	20	14		16.5
8	8	0		4.5

### Weibull parameterization to frame time-to-event outcome measurement error

As an alternative, we propose framing time-to-event outcome measurement error according to the Weibull distribution.1We consider the following parameterization of the Weibull PDF: *f*(*x*)=(*a*/*σ*) (*x*/*σ*)^*a*−1^exp(−(*x*/*σ*)^*a*^). Consider the following models of the true and mismeasured event times:

$$\log\left(Y\right)=a_{0}+\sigma \varepsilon$$

$$\text{log}\left(Y^{*}\right)=a_{0}^{*}+\sigma^{*}\varepsilon$$

where *a*_0_ is the Weibull regression intercept (i.e., log - scale), 1/*σ* is the Weibull shape, *ɛ* follows an extreme value distribution, and asterisks indicate mismeasured versions of these parameters [22]. The difference between the true and mismeasured event times can be described as:

$$\delta =\text{log}\left(Y^{*}\right)-\log\left(Y\right)=\left(a_{0}^{*}-a_{0}\right)+\left(\sigma^{*}-\sigma \right)\varepsilon$$

Here, the bias due to measurement error, *δ*, is attributed to differences in the intercept (log-scale) and shape parameters, which describe the failure event rate and its constancy (or lack of) over time, respectively. Note that assuming measurement error on the log-additive scale for time-to-event outcomes has similarities to previously established literature on multiplicative measurement error models for covariates, where mismeasured covariates may be log-Normally transformed, though the correction methods do not naturally extend as the target parameter of interest differs [6].

If measurement error exists between Y and Y* such that it affects the failure event rate, using Y* to estimate the shape and intercept (and consequentially, the median time to event or other percentiles of the CDF), will yield biased estimates for the true parameters. Furthermore, this translates to biased estimates of summary measures of interest like median time to event. To illustrate how so, we can derive the bias in median times as the ratio of medians between the true and mismeasured models, notated as *θ* and θ*, respectively:

$$\begin{array}{ll} \frac{\theta }{\theta^{*}} & =\frac{\exp\left\{\alpha \right\}\log{\left(2\right)}^{\frac{1}{\sigma }}}{\exp\left\{\alpha^{*}\right\}\log{\left(2\right)}^{\frac{1}{\sigma^{*}}}}\\ & =\exp\left(\alpha -\alpha^{*}\right)\log{\left(2\right)}^{\frac{1}{\sigma }-\frac{1}{\sigma^{*}}}\\ & \propto \exp\left(bias_{intercept}\right)\log{\left(2\right)}^{bias_{shape}} \end{array}$$

where

$$\begin{array}{cc} \text{bia}{\text{s}}_{intercept} & =\alpha -\alpha^{*}\\ \text{bia}{\text{s}}_{shape} & =\sigma -\sigma^{*} \end{array}$$

When both the shape and intercept parameters are *under-estimated* in the mismeasured outcome data (such that their biases are both negative), then θ* will be biased towards shorter median time. Conversely, when both parameters are *over-estimated* (such that their biases are both positive), then θ* will be biased towards longer median time. However, when bias in the parameters are in *opposing* directions, then θ* may appear closer to *θ* due to a potential ‘canceling out’ effect, and it is therefore important to consider the bias in the parameter estimates in order to contextualize biases in median times.

In routine clinical practice, it is indeed plausible for time-to-event outcomes to be mismeasured, resulting in biased Weibull regression parameter estimates. Consider the composite endpoint of PFS, defined by the earliest occurrence of progression or death, censored by loss to follow-up, end of study or data cut-off. If progression events are misclassified such that true events are not captured (i.e., false negatives), and PFS is either defined by a subsequent progression or death or censoring, this can lead to a mismeasured Y* that appears to have a lower event rate than the true Y, potentially resulting in mismeasured shape and intercept parameters that are greater than the truth. Conversely, erroneous and premature capture of progression events (i.e., false positives) may lead to higher event rates at earlier times, biasing the mismeasured shape and intercept in the opposing direction. We will revisit this example further in our illustrative data application.

### Survival regression calibration (SRC)

As an extension to standard regression calibration, we describe an approach to model and mitigate the measurement error bias in time-to-event outcomes by calibrating the Weibull model parameters. First, we fit a Weibull regression model using the mismeasured outcome in the full sample (denoted with subscript ‘f’) to obtain initial estimates of the mismeasured shape and intercept parameters, ${\hat{\alpha }}_{0,f}^{*}$ and ${\hat{\sigma }}_{f}^{*}$:

$$\log\left(Y_{f}^{*}\right)={{\hat{\alpha }}^{*}}_{0,f}+{\hat{\sigma }}_{f}^{*}\epsilon$$

Next, using a validation sample (denoted with subscript ‘v’), in which the true and mismeasured outcomes are both collected, we fit separate Weibull models to two outcome versions and estimate their respective shape and intercept parameters:

$$\text{log}\left(Y_{v}^{*}\right)={{\hat{\alpha }}^{*}}_{0,v}+{\hat{\sigma }}_{v}^{*}\epsilon$$

$$\log\left(Y_{v}\right)={\hat{\alpha }}_{0,v}+{\hat{\sigma }}_{v}\epsilon$$

Using the parameter estimates of the true and mismeasured intercepts and shapes, we then estimate the bias of each respective parameter:

$${\hat{bias}}_{intercept}={\hat{\alpha }}_{0,v}-{{\hat{\alpha }}^{*}}_{0,v}$$

$${\hat{bias}}_{shape}={\hat{\sigma }}_{v}-{\hat{\sigma }}_{v}^{*}$$

We calibrate the mismeasured shape and intercept in the full sample, ${\hat{\alpha }}_{0,f}^{*}$ and ${\hat{\sigma }}_{f}^{*}$, and use the calibrated Weibull parameters to construct an adjusted survival curve, which can be used to estimate the survival probabilities at time t:

$$\hat{S}\left(t\right)=\exp\;\left\{-\left(\frac{t}{\exp\left({{\hat{\alpha }}^{*}}_{0,f}+{\hat{bias}}_{intercept}\right)}\right)\;\hat{\;}\left({\hat{\sigma }}_{f}^{*}+{\hat{bias}}_{shape}\right)\right\}$$

Finally, since the median time is of interest here, we estimate the corrected median time (${\hat{\theta }}_{src}$) to event as follows:

$${\hat{\theta }}_{src}=\exp\left({{\hat{\alpha }}^{*}}_{0,f}+{\hat{bias}}_{intercept}\right)\times \log\left(2\right)\;\hat{\;}\left\{\frac{1}{{\hat{\sigma }}_{f}^{*}+{\hat{bias}}_{shape}}\right\}$$

#### Model assumptions and transportability

There are several assumptions related to the parametric modeling and validation sampling that are important to highlight. First, when fitting a Weibull accelerated failure time model, a typical assumption is that censoring is either not present or is non-informative. Next we assume that the hazard is constant, such that the sigma parameter does not vary over time. These assumptions are required when fitting the parametric models to the true and mismeasured outcomes in the approach outlined above.

Additionally, recall that in order to implement these methods to correct for outcome measurement error, a validation sample, defined as a study (or subgroup of a study) with the true and mismeasured outcomes both collected, must exist. When estimating the bias in the parameters from a validation sample, we assume that the bias estimates are transportable from the validation sample to the full study [23]. In other words, we assume that $\alpha_{0,v}-\alpha_{0,v}^{*}=\alpha_{0,f}-\alpha_{0,f}^{*}$and $\sigma_{v}-\sigma_{v}^{*}=\sigma_{f}-\sigma_{f}^{*}$, such that estimates of the differences between the true and mismeasured Weibull parameters in the validation sample are unbiased estimates of their respective differences in the full study population. When the validation sample is *internal*, and more specifically, when it is either a simple random sample or a probabilistic sample of the full study, this assumption is plausible to make. However, when the validation sample is *external,* or an internal validation sample is identified via non-probability sampling (e.g., patients are included in a validation sample based on convenience factors like geographic proximity, participation willingness, or ease of data availability to the researcher), it is possible that transportability could be violated. More specifically, if the amount of measurement error bias is differential with respect to baseline characteristics, and if the validation sample demographics differ from the main study with respect to those characteristics, then additional bias may be present, and additional adjustments may be required [24]. In the remainder of the manuscript, we will assume a representative internal validation sample is available for use such that transportability holds from the internal validation sample to the broader full sample, and we will discuss this potential challenge in the conclusion.

## Simulation study

We now present a simulation study to evaluate our proposed survival regression calibration methodology. This simulation is motivated by a case study in NDMM (described further in the Data Application), where the primary (and potentially mismeasured) endpoint of interest in a study is PFS.

### Simulation design

We simulate n=365 patients and generate true and mismeasured PFS times according to Weibull distributions. We assume a true median PFS (mPFS) of 34.2 months, and a study duration of 60 months (See Supplementary Materials for simulation results with shorter and longer true mPFS times). Patients who do not have a simulated PFS event by study completion are censored, and the censoring rate is approximately 30 %. We vary the true Weibull shapes to be either 0.8, 1, or 1.2. The true Weibull regression intercepts are optimized accordingly to preserve the desired true mPFS time and shape. To assess how the method performs under differing amounts of measurement error bias, we vary the mismeasured shape and intercept parameters to be +/− (0, 0.1, 0.3) of the true values. In other words, we simulate scenarios where (1) no measurement error is present, (2) measurement error leads to increases in the shape only, (3) measurement error leads to increases in the intercept, and (4) measurement error leads to changes in both parameters, either in similar or opposing directions.

For each simulated study sample, we randomly allocate 40 % of subjects (n≈150) to belong to an internal validation sample, in which we collect both the true and mismeasured outcomes. Note that for evaluation purposes, we retain the true outcomes in the full simulated sample; however, we fit the models to estimate the bias calibration parameters in the validation sample only. Additional simulations in which the size of the validation sample is varied can be found in the Supplementary Materials.

We compare three different estimates of mPFS in the study: (1) unadjusted, (2) adjusted via regression calibration (RC), where we assume an additive error structure and fit a linear model to estimate the bias, and (3) adjusted via survival regression calibration (SRC), where we fit Weibull regression models to estimate the bias in the shape and intercept. To assess additional sensitivity to model misspecification, we explore a fourth model where we estimate measurement error bias parameters using a log-logistic model rather than a Weibull model (further details available in the Supplementary Materials). We report bias in mPFS as the difference between the mismeasured/calibrated mPFS and the true mPFS. We run 1,000 simulation iterations, and report mPFS estimates as the mean across iterations, and we report bootstrapped confidence intervals for each type of estimated mPFS as the 2.5th and 97.5th percentiles of the distributions for the simulated estimates [25]. Coverage of the 95 % confidence interval, defined as the proportion of simulation iterations where mPFS confidence intervals contain the true mPFS value, is also estimated via the bootstrap approach, and detailed results are reported in the Supplementary Materials.

### Simulation results

We now present results from the simulation study. For simplicity, we focus on results of bias where the true Weibull shape is 1 (corresponding to an exponential distribution); additional simulation results, with similar findings, can be found in the Supplementary Materials.

#### No measurement error

When the shape and intercept parameters are equal between the true and mismeasured PFS, the bias in the unadjusted estimate is negligible on average, as expected, though in rare cases non-negligible bias remains (mPFS bias=0.14 months, 95 % CI=−6.2 to 6.27 months). Both the RC and SRC-adjusted estimates have slightly less bias, with comparable amounts of variability (RC-adjusted mPFS bias=0.08 months, 95 % CI=−5.43 to 5.45 months; SRC-adjusted mPFS bias=0.2 months, 95 % CI=−4.98 to 6.1 months).

#### Measurement error in the Weibull intercept parameter

**Figure 1:**
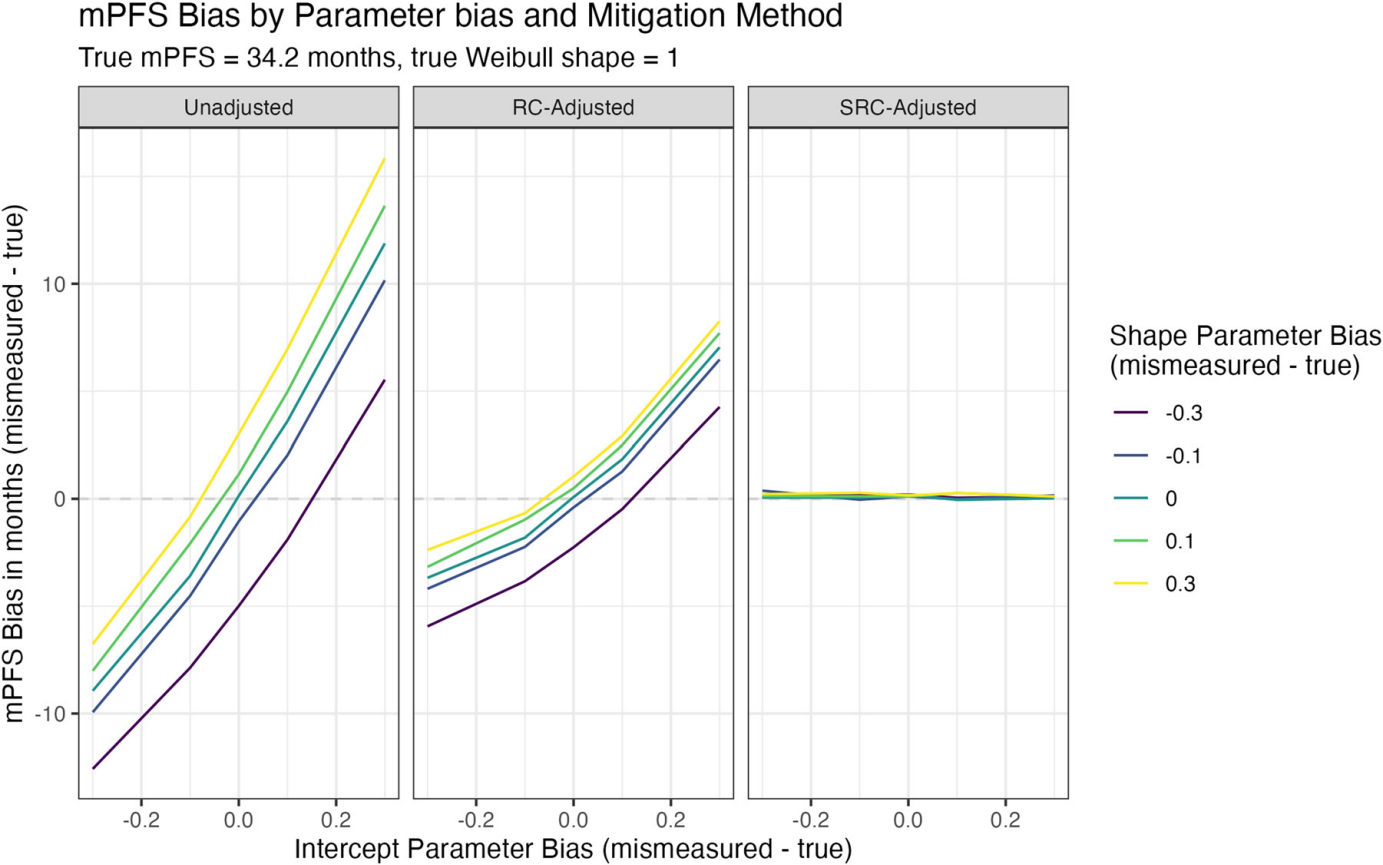
Simulation results when true Weibull shape=1. The left panel shows the observed mPFS bias when varying bias in the intercept (by x axis) and bias in the shape (by color). The middle panel shows bias after applying standard regression calibration methods. The right panel shows bias after applying the proposed survival regression calibration method.

When measurement error leads to differences in the intercept parameter only (i.e., only the intercept parameter is different due to measurement error), but the shape parameters are equivalent (Figure 1, left panel, turquoise line), such differences in the intercept can lead to unadjusted mPFS that is anywhere from a year greater than to ∼9 months less than the true mPFS. More specifically, unadjusted mPFS is longer than the true mPFS when measurement error leads to larger intercepts (e.g., unadjusted mPFS bias when true intercept is 3.9 and mismeasured intercept is 4.2: 11.88 months, 95 % CI=4.86 to 19.59 months). Unadjusted mPFS is shorter than the true mPFS when measurement error leads to smaller intercepts (e.g., unadjusted mPFS bias when true intercept is 3.9 and mismeasured intercept is 3.6: −8.94 months, 95 % CI=−14.25 to −3.66 months). Linear regression calibration (RC) can reduce this bias, albeit not completely (RC-adjusted mPFS bias ranges from −3.7 to 7 months, Figure 1, middle panel, turquoise line). Survival regression calibration (SRC) reduces the bias to a greater extent than RC, such that the estimates of SRC-adjusted mPFS bias, under intercept parameter error, range −0.05 to 0.06 months (Figure 1, right panel, turquoise line).

#### Measurement error in the Weibull shape parameter

When measurement error leads to differences in the shape parameter only (i.e., the true and mismeasured intercepts equal 3.9, Figure 1 left panel, x axis=0), such differences can lead to unadjusted mPFS bias that ranges from −5 to 3 months. Unadjusted mPFS is biased towards earlier times when the mismeasured shape is less than the true shape (i.e., unadjusted mPFS bias when true shape is 1 and mismeasured shape is 0.7: −4.99 months, 95 % CI = −11.19 to 1.51 months), and biased towards later times when the mismeasured shape is greater than the true shape (i.e., unadjusted mPFS bias when true shape is 1 and mismeasured shape is 1.3: 3.01 months, 95 % CI=−2.77 to 8.55 months). Here, RC again reduces the bias partially (RC-adjusted mPFS bias ranges from −2.26 to 1.04 months, Figure 1 middle panel, x axis=0), but not to the same magnitude as SRC (SRC-adjusted mPFS bias ranges from 0.1 to 0.2 months, Figure 1 right panel, x axis=0).

#### Measurement error in both parameters

When measurement error leads to differences in *both* Weibull parameters in similar directions (i.e., both parameters are greater than, or less than, the true respective parameters), it can amplify the amount of resulting bias in a nonlinear fashion (See Table 2). In such cases, SRC continues to outperform RC in bias reduction. For instance, when mismeasured shape and intercept are both 0.3 less than the true values, RC-adjusted mPFS bias is still −5.94 months (95 % CI=−11.07 to −0.74 months) while SRC-adjusted mPFS bias is 0.08 months (95 % CI: −4.97 to 5.49 months). When both mismeasured parameters are 0.3 *greater* than the true values, RC-adjusted mPFS bias is 8.26 months (95 % CI=2.45 to 14.28 months) while SRC-adjusted mPFS bias is 0.11 months (95 % CI: −4.44 to 5.38 months).

**Table 2:** Simulation results when true shape=1 (i.e., true data come from exponential distribution).

True shape	Mismeasured shape	True intercept	Mismeasured intercept	Observed mPFS bias (95 % CI)	RC-adjusted mPFS bias (95 % CI)	SRC-adjusted mPFS bias (95 % CI)
No measurement error						
1	1.0	3.90	3.90	0.14 (−6.2, 6.27)	0.08 (−5.43, 5.45)	0.2 (−4.98, 6.1)
**Measurement error in intercept only**						
1	1	3.90	3.60	−8.94 (−14.25, −3.66)	−3.68 (−8.69, 1.28)	0.06 (−4.74, 5.44)
			3.80	−3.59 (−9.42, 2.13)	−1.81 (−7.14, 3.3)	0.06 (−4.85, 5.16)
			4.00	3.6 (−2.31, 10.21)	1.84 (−3.85, 7.44)	−0.05 (−5.29, 5.74)
			4.20	11.88 (4.86, 19.59)	7.04 (1.11, 13.91)	0.03 (−4.69, 6.21)
**Measurement error in shape only**						
1	0.7	3.90	3.90	−4.99 (−11.19, 1.51)	−2.26 (−7.99, 4.07)	0.2 (−5.02, 5.76)
	0.9			−1.05 (−6.87, 5.02)	−0.4 (−5.89, 4.89)	0.11 (−4.73, 5.86)
	1.1			1.14 (−4.87, 7.3)	0.49 (−5.22, 5.7)	0.1 (−4.8, 5.66)
	1.3			3.01 (−2.77, 8.55)	1.04 (−3.85, 6.38)	0.16 (−4.95, 5.89)
**Measurement error in both parameters: both smaller than truth**						
1	0.7	3.90	3.60	−12.58 (−18.16, −6.84)	−5.94 (−11.07, −0.74)	0.08 (−4.97, 5.49)
			3.80	−7.87 (−14.11, −1.56)	−3.84 (−10.08, 1.96)	0.17 (−5.11, 5.6)
	0.9		3.60	−9.94 (−15.41, −4.77)	−4.19 (−9.01, 0.61)	0.38 (−4.69, 6.07)
			3.80	−4.52 (−10.82, 1.45)	−2.24 (−7.59, 2.75)	−0.04 (−4.78, 5.21)
**Measurement error in both parameters: both larger than truth**						
1	1.1	3.90	4.00	4.97 (−0.81, 10.8)	2.49 (−2.83, 8.04)	0.27 (−4.59, 6)
			4.20	13.63 (6.52, 21.54)	7.7 (1.57, 13.86)	0.11 (−4.71, 5.52)
	1.3		4.00	6.96 (1.36, 13.25)	2.91 (−2.69, 8.31)	0.27 (−5.03, 5.98)
			4.20	15.85 (9.66, 22.46)	8.26 (2.45, 14.28)	0.11 (−4.44, 5.38)
**Measurement error in both parameters: shape smaller, intercept larger than truth**						
1	0.7	3.90	4.00	−1.9 (−8.79, 5.41)	−0.48 (−6.91, 6.1)	0.05 (−5.11, 5.75)
			4.20	5.53 (−2.51, 14.12)	4.26 (−3.48, 12.33)	0.11 (−5.23, 5.74)
	0.9		4.00	2.03 (−4.2, 8.26)	1.27 (−4.78, 7.9)	−0.01 (−4.86, 5.53)
			4.20	10.16 (2.56, 18.94)	6.47 (0.17, 14.43)	0.16 (−4.83, 5.5)
**Measurement error in both parameters: shape larger, intercept smaller than truth**						
1	1.1	3.90	3.60	−8.01 (−13.13, −3.23)	−3.17 (−7.83, 1.5)	0.13 (−4.92, 5.68)
			3.80	−2.08 (−7.5, 3.33)	−0.97 (−6.2, 3.97)	0.12 (−5.09, 5.57)
	1.3		3.60	−6.77 (−11.47, −2.32)	−2.38 (−7.53, 2.01)	0.22 (−4.67, 6.1)
			3.80	−0.84 (−6.37, 4.35)	−0.67 (−5.43, 3.96)	0.28 (−5.05, 6.11)

Conversely, measurement error that leads to an increase in one parameter, but a decrease in the other, could result in a scenario with no observed bias. For example, when the mismeasured shape is 1.3 (greater than true shape of 1.0) and the mismeasured intercept is 3.8 (smaller than the true intercept of 3.9), the unadjusted mPFS bias is −0.84 months (95 % CI: −6.37 to 4.35 months). In other words, it is possible that biases due to differences in these Weibull parameters may counteract one another if they appear in opposing directions, leading to an observed and unadjusted mPFS that is unbiased. In the lefthand panel of Figure 1, this can be further observed by noting where the different-colored lines (representing different amounts of shape bias) cross the dotted line of 0 at different points along the x axis (representing different amounts of intercept bias). While adjustment with SRC still yields unbiased mPFS estimates (mPFS bias=0.28 months, 95 % CI: −5.05 to 6.11 months), this is an important cautionary finding to study these biases carefully and recognize that unadjusted mPFS that appears ‘unbiased’ may not necessarily mean the outcome is error-free.

#### 95 % confidence interval coverage

Broadly, measurement error leads to poor coverage for the unadjusted mismeasured mPFS estimate, RC demonstrates improved coverage under minimal amounts of measurement error, and SRC yields consistently high confidence interval coverage. This is, in part, due to greater variability of mPFS estimates when applying SRC, and is also associated with the size of the simulated internal validation sample. Smaller validation samples yield greater variability, and confidence interval coverage closer to 100 %. More detailed findings on 95 % confidence interval coverage can be found in the Supplementary Materials.

## Data application

We next apply the developed SRC method to an illustrative example in NDMM. Disease assessments in multiple myeloma (MM) are conducted according to standards by the International Myeloma Working Group (IMWG) Uniform Response Criteria based on imaging and biomarkers from blood, urine, and bone marrow biopsy testing [26]. In MM clinical trials, assessments are conducted rigorously, per strict protocol. In routine clinical practice, however, the adherence to practice guidelines like IMWG may be less strict compared to a trial, and as a result, MM endpoints in RWD may be derived with error relative to trial standards. For example, MM trials typically require collection of serum and urine labs every 28 days, while in routine clinical practice, it is more common for only serum to be collected, and on a more variable assessment frequency. This variability in measurement can introduce bias when comparing outcomes between RWD and RCTs. With this motivation in mind, we illustrate the application of our methodology to estimate mPFS in the control arm of the MAIA trial, in which patients with NDMM were treated with lenalidomide and dexamethasone (Rd) [27]. Note that, by using data from a completed trial and constructing an endpoint that is analogous to those collected in routine practice, we are able to illustrate how this methodology mitigates bias due to measurement error without concern of other biases common in studies using RWD being present (i.e., selection bias, surveillance bias).

For this exercise, we assume the trial-collected outcomes are measured without error, and will constitute our ‘true’ outcomes. We derive ‘mismeasured’ outcomes by applying a subset of IMWG criteria to the trial data per real-world standards [28]. While these criteria are *similar* to the full IMWG criteria, it is possible that a less strict approach to progression capture in clinical practice could yield either false positive progression events, false negatives, or both.

To emulate the availability of an internal validation sample, we randomly sample 40 % of the trial control arm, for whom we ‘collect’ both the true and mismeasured progression events. In practice, the existence of such validation samples may not be feasible for studies utilizing RWD in certain therapeutic areas, hence the illustrative nature of this example. We perform a double-bootstrap procedure to estimate variance of the mPFS, sampling the control arm population as well as the internal validation sample with replacement, over 1,000 iterations. Confidence intervals are constructed by reporting the 2.5th and 97.5th percentiles of the mPFS estimate distribution across the 1,000 re-samplings. We compare the true trial estimates, the mismeasured ‘real-world’ estimates, and the survival regression-calibrated estimates of mPFS to one another.

## Results

First, note that bias exists due to measurement error between the trial and ‘real-world’ endpoint versions in the full study sample. The ‘real-world’ (rw) mPFS appears to be biased towards earlier times (mPFS bias=−2.6 months), likely due to high false positive rates in classifying progression events per the flexible criteria. When comparing the Weibull model parameters between the trial and ‘real-world’ outcomes, the ‘real-world’ shape and intercept are both smaller than the trial parameter estimates (Table 3). This further highlights that both parameters, in practice, may be mismeasured, as opposed to just one or the other, and it is therefore important to calibrate both parameters in the model.

**Table 3:** True (trial) and mismeasured rwPFS and Weibull parameter estimates in full study sample.

Outcome version	mPFS, months	Weibull shape	Weibull intercept (log-Weibull scale)
‘True’ trial PFS	34.8	0.97	3.92
‘Mismeasured’ rwPFS	32.2	0.92	3.86

**Figure 2:**
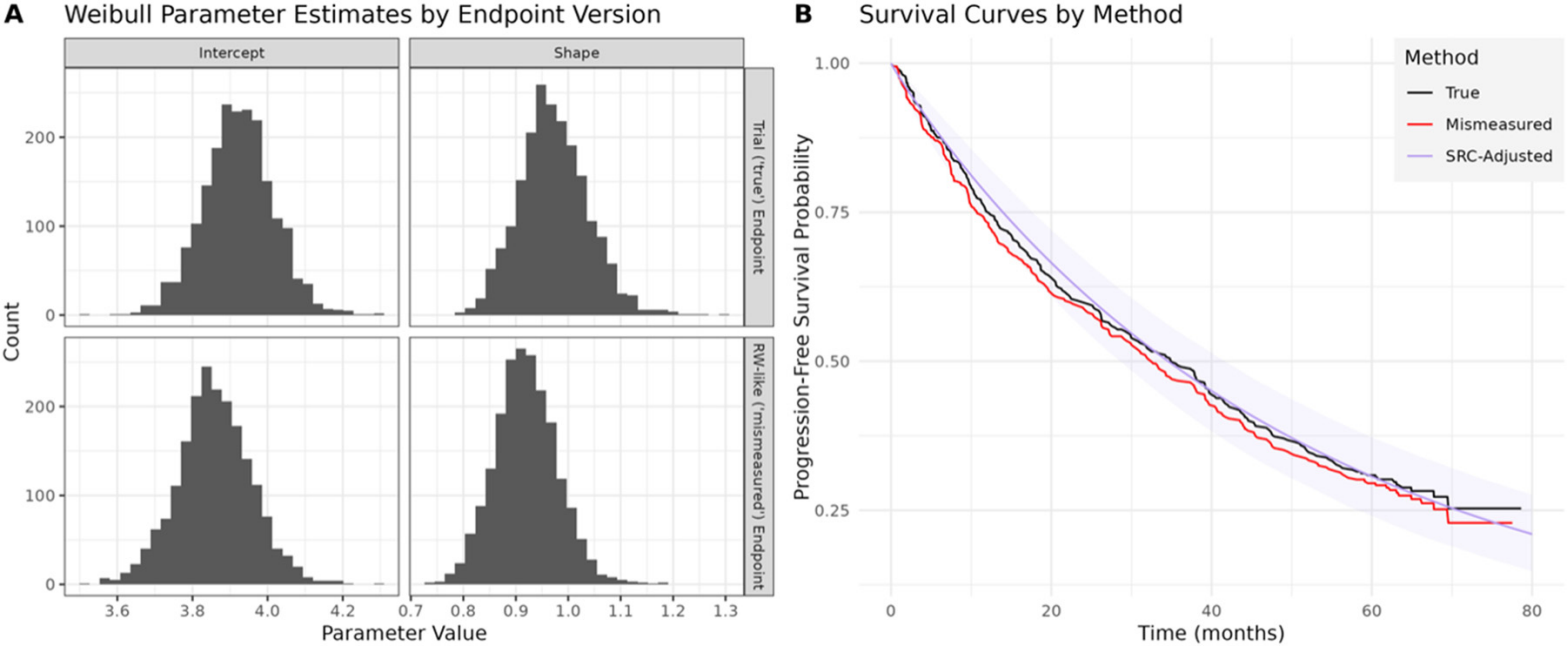
Results from the illustrative data application. A) Weibull parameter estimates by endpoint version, B) survival curves (true and mismeasured), along with survival regression calibration-adjusted estimates.

Figure 2a shows the estimates of the trial and ‘real-world’ Weibull parameters in the bootstrapping validation sample, which are used to adjust for this bias in the ‘real-world’ PFS among all patients in the full sample. Similar to Table 3, these bootstrapping estimates of the shape and intercept parameters in the ‘real-world’ outcome model are *smaller* on average than the ‘truth’ in the trial outcome model. Figure 2b compares the true and mismeasured PFS curves to the SRC-adjusted curve. Again, in a typical study we would not observe the true (black) curve, only the mismeasured (red) curve. By retaining the data on true trial outcomes, we see the SRC method is able to reduce the bias and better estimate the true mPFS estimate (SRC-adjusted mPFS=34.5 months, 95 % CI: 28.5–41.6 months). Note that the confidence interval width is reflective of the validation sample size, as well as the overall size of the control arm.

## Discussion

Standard regression calibration methodology, which assumes a linear additive error structure on a Normally-distributed outcome, may not be suitable to mitigate measurement error for time-to-event outcomes due to fundamental model misspecification and risk of generating implausible (i.e., negative) event times. In this paper, we proposed a survival regression calibration (SRC) method that frames time-to-event outcome measurement error as the difference in Weibull parameters and accordingly calibrates bias in each parameter. Whereas previous regression calibration methods could yield negative event times and fail to account for event misclassification, our SRC method is better suited for time-to-event outcomes. Through simulation, we demonstrated that SRC not only outperforms regression calibration across a range of existing measurement error biases, but also successfully mitigates measurement error bias when present. We then illustrated the use of SRC in a data example to correct for error in real-world PFS in a cohort with NDMM.

There are several takeaways, limitations, and opportunities for future work to highlight. First, our developed approach can potentially address measurement error bias when estimating a marginal summary quantity, like mPFS, within a real-world study population. Furthermore, we explore a case where any amount of outcome measurement error is consistent across patient subgroups. This may be particularly relevant when constructing an external benchmark using RWD to contextualize single-arm trial results, in which indirect comparisons are typically drawn between real-world and trial outcomes. When treatment effect estimates are relevant to the research question, whether marginal or conditional on baseline covariates, additional research is needed to extend the calibration of outcomes when estimating hazard ratios and other relative effect measures. When measurement error in outcomes is differential by baseline characteristics, future work should also evaluate the plausibility of utilizing conditional models to estimate the shape and intercept bias parameters. Such differential error may also contribute to informative censoring, and should thus be studied further. Nevertheless, the direction and impact of these biases at the study population level, whether estimating mPFS or a hazard ratio, are related, and thus our methods can still be impactful for broader inferences. For example, when rwPFS is biased towards longer times, assuming no other bias or confounding, a hazard ratio comparing such real-world and trial PFS would bias towards the null, as it would make an experimental trial treatment appear less efficacious by comparison.

Similarly, we have developed and evaluated this method to address measurement error when it is present, on average, for an entire real-world comparator cohort. While it is possible that error may be more pronounced for certain patient subgroups defined by baseline characteristics, our work is easily extendable to stratified analyses, such that bias can be addressed within each patient strata of interest. Future work may also consider extending to more complex error structures, such as those that depend on covariates, as well as to other parametric survival models [29].

Next, we demonstrated via simulation that our method can mitigate measurement error bias by estimating both the true and mismeasured Weibull regression parameters in a validation sample. In practice, validation samples can be challenging to conceptualize or curate for progression and response-based endpoints, particularly when using retrospective RWD where outcomes may be abstracted from unstructured clinical documents or derived from diagnostic codes. For solid tumor diseases in oncology, where imaging-based endpoints are commonly used in clinical trials, there may be opportunity to curate validation samples among real-world patients who have imaging data available, which could be processed and segmented independently and compared to abstracted ‘mismeasured’ outcomes. For retrospective MM RWD, however, validation samples may be more challenging to design in practice for these outcomes. Notably, patterns of IMWG lab observability and the use of flexible IMWG algorithms to derive response outcomes may make it infeasible to design or identify an internal validation sample where ‘trial-like’ outcomes are measurable according to full IMWG algorithmic criteria. For endpoints like overall survival, where RWD capture of death events may be imprecise, data linkages to registries and other reliable external data sources can enable modeling and mitigation of measurement error bias [30]. Hybrid control arm designs, where a randomized trial’s comparator arm is augmented by RWD, may also produce valuable validation data required for outcome calibration [31]. Thoughtful study planning and prospective data capture from routine practice settings can further help address these challenges, and additional research on innovative study design is critical alongside the development of these methods.

We focused on the case where an *internal* validation sample is available, such that the necessary modeling to mitigate measurement error bias can be performed in a direct (and randomly or probabilistically sampled) subset of the RWD cohort of interest. By doing so, we have assumed that bias parameter estimates are transportable from a validation sample to the full study sample. When using an external validation sample for method application (i.e., using a separate patient set from the cohort of interest), transportability becomes a larger concern, namely when baseline differences exist between the primary study and external validation sample populations. It is important, when such concerns arise, to compare the populations closely and ensure similarities whenever possible, particularly on factors associated with the measurement error patterns. Future work should also explore extensions of our methods to account for transportability violations, such as using weighted estimators, or through sensitivity analyses for the calibrated Weibull parameters. Given the context-dependent challenges highlighted above when curating validation samples, it is also plausible that a validation sample may not be available at all for a given application. In the absence of appropriate validation samples, sensitivity analyses or quantitative bias analyses may be important tools to evaluate the robustness of study results to outcome measurement error, and such approaches should be further developed as well.

Lastly, when addressing outcome measurement error in non-experimental studies (such as RWE), there are often other contributing sources of bias (i.e. selection bias, immortal time bias) that must be addressed to draw rigorous and causal inferences. By assessing how bias in studies using RWD may be attributed to measurement error, we have developed an approach to address such concerns and fill a critical gap in the literature. It is imperative for future research to consider how to integrate this methodology with other methods and tools to account for other types of bias. Nevertheless, we have demonstrated that measurement error in time-to-event outcomes can cause substantial bias on its own, and we have developed improved methodology that can appropriately calibrate such mismeasured outcomes. Further development and adoption of these methods may enable more efficient and robust evidence generation in studies using RWD in drug development and ultimately improve comparability between trial and real-world findings.

## Supplementary Material

Supplementary Material Details
