# Supplementary material for: Regression calibration for time-to-event outcomes: mitigating bias due to measurement error in real-world endpoints
Source: Epidemiol Methods. 2025 Sep 26;14(1):20250009. doi: 10.1515/em-2025-0009 (PMC12464481; doi:10.1515/em-2025-0009)
Supplement: Supplementary file 1 — Supplementary Material Details [file j_em-2025-0009_suppl_001.docx]

## Supplemental Material for “Regression calibration for time-to-event outcomes: Mitigating bias due to measurement error in real-world endpoints”

### Additional Simulation Results

#### Survival Curves for Simulation Results (when true shape = 1)

Figure S1 shows survival curves for nine of the simulation scenarios varying the amount of intercept and shape parameter bias (when true shape = 1). All 1,000 simulated curves are plotted in faint colors, with the average survival probabilities shown in bolder lines. The unadjusted mismeasured curves are in yellow, RC-adjusted curves in purple, and SRC-adjusted curves in turquoise. The true median PFS is denoted at the intersection of the dotted grey lines. Note that while standard regression calibration may reduce bias in the *median* PFS time, the RC-adjusted curves often over- (or under-) estimate survival probabilities, namely at earlier times. For example, when the intercept is biased by -0.3 (Figure S1-D, F, and I), standard regression calibration over-corrects the survival probabilities at the beginning of the distribution. This is likely due to the generation of negative calibrated times, which do not contribute to the survival function.

###
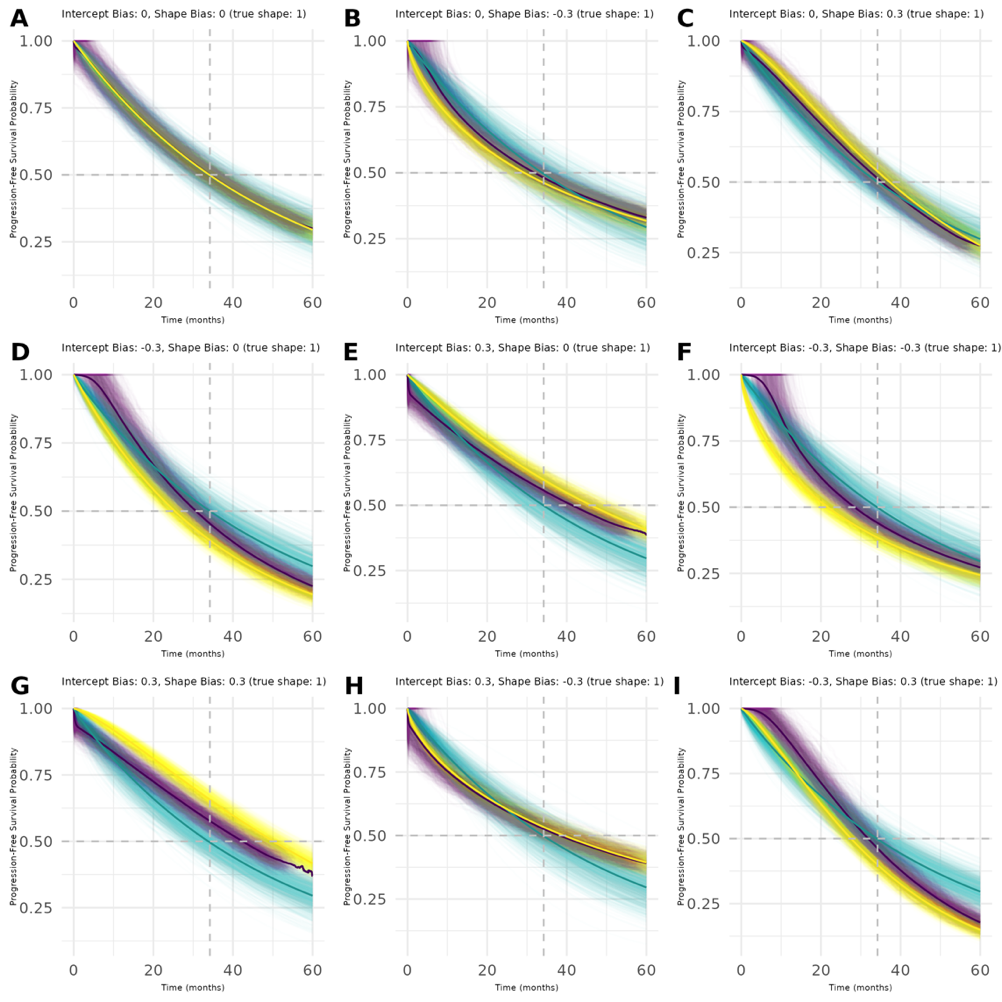


Figure S1: Simulated survival curves by intercept bias, shape bias, and method. Yellow = unadjusted mismeasured PFS, purple = RC-adjusted PFS and turquoise = SRC-adjusted PFS. Plot A = no bias, B & C = shape bias only, D & E = intercept bias only, F-I = intercept and shape bias

#### Varying Validation Sample Size

In the simulation study presented, we assumed that 40% of the 365 patients (N = 146) were sub-sampled for an internal validation study, such that true and mismeasured outcomes could be used to model and mitigate measurement error bias. In order to evaluate the impact of the validation sample size further, we varied the proportion sampled for internal validation to be smaller (10%, N ~ 37) and larger (90%, N ~ 329). Figure S2 shows how confidence interval widths of the median PFS bias estimates vary by validation sample size and method. Each line represents a different simulation scenario (all combinations of true/observed shape and intercept parameter). When validation samples are larger, we are able to mitigate measurement error bias more precisely (i.e., the confidence interval widths are smaller); however, when validation samples are small, estimates of the bias parameters may be more variable (i.e. the confidence interval widths are larger).


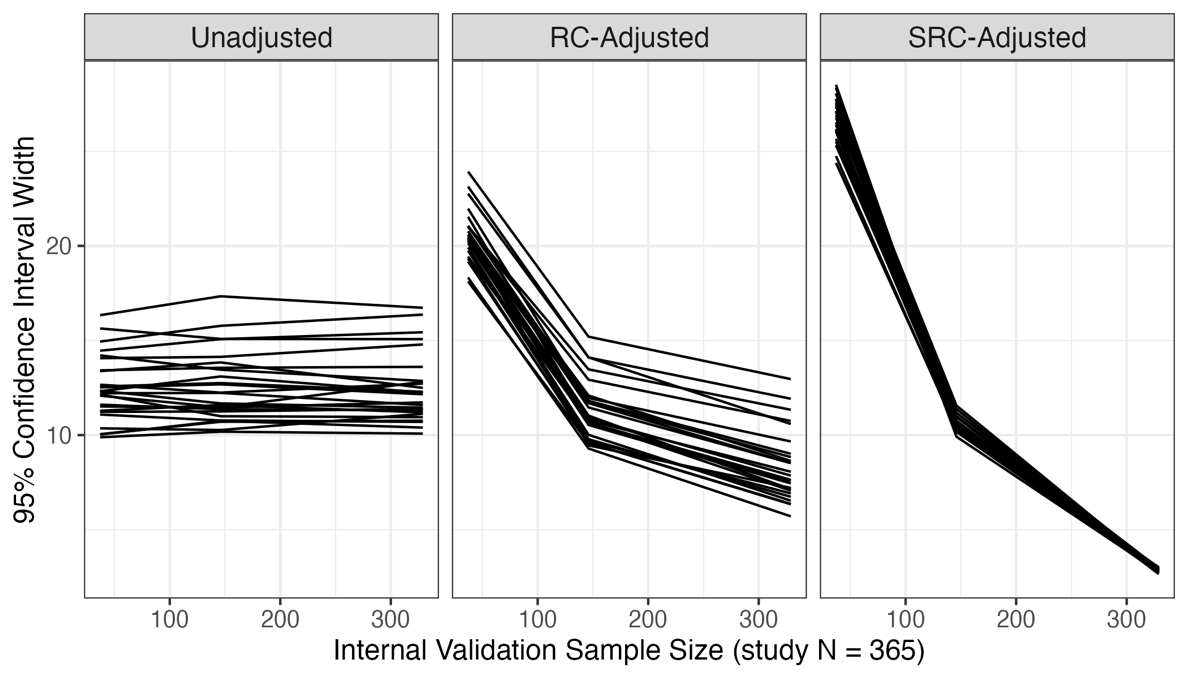


Figure S2: Bootstrapped 95% Confidence Interval Width (97.5^th^ quantile – 2.5^th^ quantile) of median PFS bias estimates, by method, and by number of patients sampled for the validation study.

#### Varying True Shape Parameter

Table S1 and Table S2 show the simulation results when the true shape parameters are 0.8 and 1.2, respectively. The results are similar to when the true shape parameter is 1 – SRC performs best in reducing bias between true and mismeasured median PFS.

Table S1: Simulation Results when True Shape = 0.8

| **True Shape** | **Mismeasured Shape** | **True Intercept** | **Mismeasured Intercept** | **Observed mPFS Bias (95% CI)** | **RC-adjusted mPFS Bias (95% CI)** | **SRC-adjusted mPFS Bias (95% CI)** |
| --- | --- | --- | --- | --- | --- | --- |
| No measurement error | | | | | | |
| 0.8 | 0.8 | 4.0 | 4.0 | -0.18 (-7.21, 7.4) | -0.17 (-6.63, 6.66) | 0.07 (-6.12, 7.32) |
| Measurement Error in intercept only | | | | | | |
| 0.8 | 0.8 | 4.0 | 3.7 | -8.87 (-16.18, -2.51) | -4.51 (-10.29, 1.12) | 0.21 (-5.96, 7.12) |
|  |  |  | 3.9 | -3.34 (-10.65, 3.9) | -1.95 (-8.27, 4.43) | 0.15 (-5.93, 7.51) |
|  |  |  | 4.1 | 3.68 (-4.53, 11.47) | 2.12 (-4.47, 8.81) | 0.13 (-6.26, 7.51) |
|  |  |  | 4.3 | 12.23 (2.9, 21.6) | 7.92 (-0.48, 16.61) | 0.11 (-6.15, 7.65) |
| Measurement error in shape only | | | | | | |
| 0.8 | 0.5 | 4.0 | 4.0 | -8.3 (-16.37, 0.88) | -4.72 (-12.17, 3.47) | 0.19 (-6.1, 7.89) |
|  | 0.7 |  |  | -2.09 (-10.43, 6.56) | -1.07 (-7.98, 6.14) | 0.32 (-5.62, 7.77) |
|  | 0.9 |  |  | 1.6 (-6.11, 9.22) | 0.53 (-6.44, 7.06) | 0.23 (-6.47, 8.05) |
|  | 1.1 |  |  | 4.52 (-2.85, 11.63) | 1.73 (-4.15, 8.09) | 0.15 (-6.05, 7.67) |
| Measurement error in both parameters: both smaller than truth | | | | | | |
| 0.8 | 0.5 | 4.0 | 3.7 | -14.82 (-22, -7.94) | -8.15 (-14.69, -1.58) | 0.28 (-6.2, 7.79) |
|  |  |  | 3.9 | -10.61 (-18.32, -1.74) | -6.01 (-13.09, 1.15) | 0.17 (-6.63, 7.67) |
|  | 0.7 |  | 3.7 | -10.51 (-17.35, -3.54) | -5.45 (-11.26, 0.77) | 0.14 (-5.82, 7.12) |
|  |  |  | 3.9 | -5.42 (-13.24, 1.71) | -2.92 (-9.14, 3.93) | 0.24 (-5.99, 7.17) |
| Measurement error in both parameters: both larger than truth | | | | | | |
| 0.8 | 0.9 | 4.0 | 4.1 | 5.59 (-1.76, 13.73) | 2.9 (-3.75, 9.94) | 0.34 (-6.15, 8.1) |
|  |  |  | 4.3 | 14.45 (5.97, 23.62) | 8.86 (0.78, 16.73) | 0.09 (-6.42, 7.71) |
|  | 1.1 |  | 4.1 | 8.67 (1.22, 16.6) | 4.2 (-2.21, 10.93) | 0.26 (-5.94, 7.54) |
|  |  |  | 4.3 | 18.03 (9.1, 27.01) | 10.23 (2.94, 16.9) | 0.22 (-6.27, 7.31) |
| Measurement error in both parameters: shape smaller, intercept larger than truth | | | | | | |
| 0.8 | 0.5 | 4.0 | 4.1 | -5.42 (-15.02, 3.98) | -2.72 (-10.64, 6.04) | 0.42 (-5.83, 8.73) |
|  |  |  | 4.3 | 0.92 (-8.91, 12.09) | 1.59 (-7.17, 11.7) | 0.14 (-6.18, 7.6) |
|  | 0.7 |  | 4.1 | 1.38 (-6.85, 10.72) | 1.17 (-6.44, 8.43) | 0.16 (-6.27, 7.22) |
|  |  |  | 4.3 | 8.91 (-0.26, 19.4) | 6.45 (-1.41, 15.51) | 0.25 (-5.92, 7.42) |
| Measurement error in both parameters: shape larger, intercept smaller than truth | | | | | | |
| 0.8 | 0.9 | 4.0 | 3.7 | -7.5 (-13.76, -0.84) | -3.91 (-9.47, 1.43) | 0.33 (-5.48, 7.76) |
|  |  |  | 3.9 | -1.44 (-8.27, 5.58) | -1.04 (-7.51, 5.62) | 0.16 (-5.85, 7.49) |
|  | 1.1 |  | 3.7 | -5.55 (-11.76, 0.45) | -2.77 (-8.23, 2.6) | 0.27 (-6.3, 8.13) |
|  |  |  | 3.9 | 0.89 (-5.56, 7.7) | -0.09 (-6.06, 6.06) | 0.28 (-5.71, 7.51) |

Table S2: Simulation Results when True Shape = 1.2

| **True Shape** | **Mismeasured Shape** | **True Intercept** | **Mismeasured Intercept** | **Observed mPFS Bias (95% CI)** | **RC-adjusted mPFS Bias (95% CI)** | **SRC-adjusted mPFS Bias (95% CI)** |
| --- | --- | --- | --- | --- | --- | --- |
| No measurement error | | | | | | |
| 1.2 | 1.2 | 3.84 | 3.84 | -0.05 (-5.01, 4.82) | 0 (-4.49, 4.73) | 0.12 (-3.89, 5) |
| Measurement Error in intercept only | | | | | | |
| 1.2 | 1.2 | 3.84 | 3.54 | -8.81 (-13.11, -4.44) | -2.73 (-7.03, 1.52) | 0.14 (-4.1, 4.42) |
|  |  |  | 3.74 | -3.17 (-7.97, 1.89) | -1.34 (-5.96, 3.05) | 0.08 (-3.93, 4.6) |
|  |  |  | 3.94 | 3.66 (-1.9, 8.7) | 1.73 (-3.29, 6.76) | 0.13 (-3.93, 4.57) |
|  |  |  | 4.14 | 11.96 (5.71, 18.27) | 6.37 (0.83, 11.92) | 0.13 (-3.77, 4.58) |
| Measurement error in shape only | | | | | | |
| 1.2 | 0.9 | 3.84 | 3.84 | -3.19 (-8.63, 2.55) | -1.21 (-6.6, 3.51) | 0.08 (-3.84, 4.58) |
|  | 1.1 |  |  | -0.97 (-5.83, 4.19) | -0.37 (-4.94, 4.58) | 0.09 (-4.07, 4.74) |
|  | 1.3 |  |  | 0.9 (-3.94, 5.49) | 0.14 (-4.35, 4.57) | -0.02 (-4.1, 4.57) |
|  | 1.5 |  |  | 2.21 (-2.43, 6.71) | 0.66 (-3.87, 5.24) | 0.14 (-4.02, 4.61) |
| Measurement error in both parameters: both smaller than truth | | | | | | |
| 1.2 | 0.9 | 3.84 | 3.54 | -11.51 (-16.23, -6.94) | -4.41 (-9.09, 0.04) | 0.16 (-3.59, 4.93) |
|  |  |  | 3.74 | -6.08 (-11.07, -0.85) | -2.5 (-7.55, 2.35) | 0.03 (-4.09, 4.5) |
|  | 1.1 |  | 3.54 | -9.55 (-14.2, -5.22) | -3.34 (-7.75, 1.15) | 0.03 (-3.98, 4.47) |
|  |  |  | 3.74 | -4.18 (-8.95, 0.76) | -1.63 (-5.94, 2.91) | 0.08 (-4.21, 4.57) |
| Measurement error in both parameters: both larger than truth | | | | | | |
| 1.2 | 1.3 | 3.84 | 3.94 | 4.41 (-0.54, 9.89) | 1.71 (-3.23, 6.46) | 0 (-4.15, 4.73) |
|  |  |  | 4.14 | 13.13 (7.14, 19.58) | 6.6 (1.29, 11.76) | 0.09 (-3.9, 4.57) |
|  | 1.5 |  | 3.94 | 5.95 (0.83, 10.82) | 2.19 (-2.33, 6.67) | 0.15 (-3.9, 4.43) |
|  |  |  | 4.14 | 14.89 (9.68, 20.74) | 7 (2.26, 11.93) | 0.04 (-3.98, 4.68) |
| Measurement error in both parameters: shape smaller, intercept larger than truth | | | | | | |
| 1.2 | 0.9 | 3.84 | 3.94 | -0.12 (-6.13, 5.66) | 0.32 (-5.17, 6.07) | 0.1 (-4.09, 4.68) |
|  |  |  | 4.14 | 7.7 (0.97, 14.6) | 5.1 (-1.29, 11.61) | 0.22 (-3.85, 4.51) |
|  | 1.1 |  | 3.94 | 2.56 (-2.72, 8.09) | 1.36 (-3.57, 6.67) | 0.04 (-4.2, 4.58) |
|  |  |  | 4.14 | 10.79 (4.31, 17.47) | 6.1 (0.46, 12.07) | -0.09 (-4.28, 4.4) |
| Measurement error in both parameters: shape larger, intercept smaller than truth | | | | | | |
| 1.2 | 1.3 | 3.84 | 3.54 | -8.29 (-12.47, -4.1) | -2.59 (-6.61, 1.63) | -0.09 (-4.34, 4.34) |
|  |  |  | 3.74 | -2.63 (-7.3, 1.96) | -1 (-5.34, 3.32) | 0.12 (-4.21, 4.83) |
|  | 1.5 |  | 3.54 | -7.44 (-11.55, -3.41) | -1.91 (-5.86, 1.98) | 0.01 (-3.87, 4.49) |
|  |  |  | 3.74 | -1.26 (-5.52, 3.35) | -0.58 (-4.96, 3.45) | 0.04 (-3.96, 4.55) |

#### Varying true median PFS to be longer or shorter than 34.2 months

We also evaluate performance of SRC compared to standard regression calibration methods when the true median PFS is either shorter or longer than 34.2 months. We consider two scenarios, where the true shape parameter is 1, but the true median PFS is either 15 months (Figure S3) or 50 months (Figure S4). Note that, in both cases, results are similar to when the true mPFS is 34.2 months. Bias in the shape and/or intercept Weibull parameters leads to bias in the mismeasured mPFS estimate. Existing regression calibration methods may reduce the bias partially, while SRC yields greater bias reduction, and therefore closer calibration of the mPFS estimates.


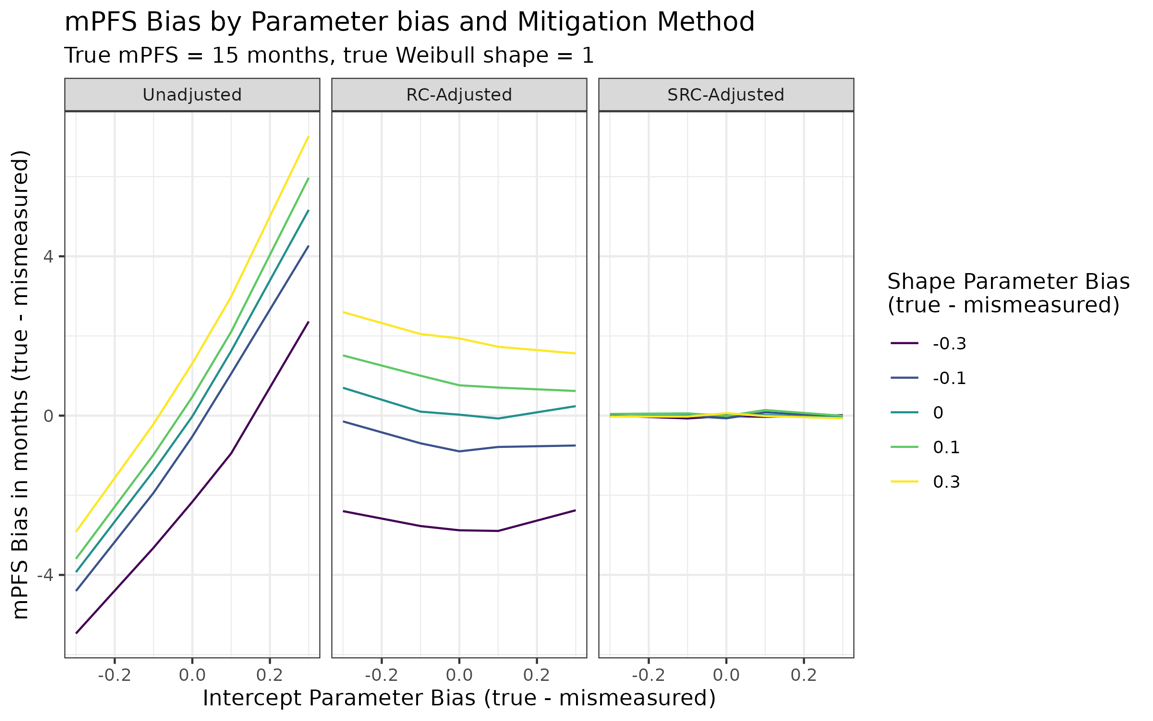


Figure S3: Simulation results when true mPFS = 15 months (Weibull shape = 1). The left panel shows the observed mPFS bias when varying bias in the intercept (by x axis) and bias in the shape (by color). The middle panel shows bias after applying standard regression calibration methods. The right panel shows bias after applying the proposed survival regression calibration method.


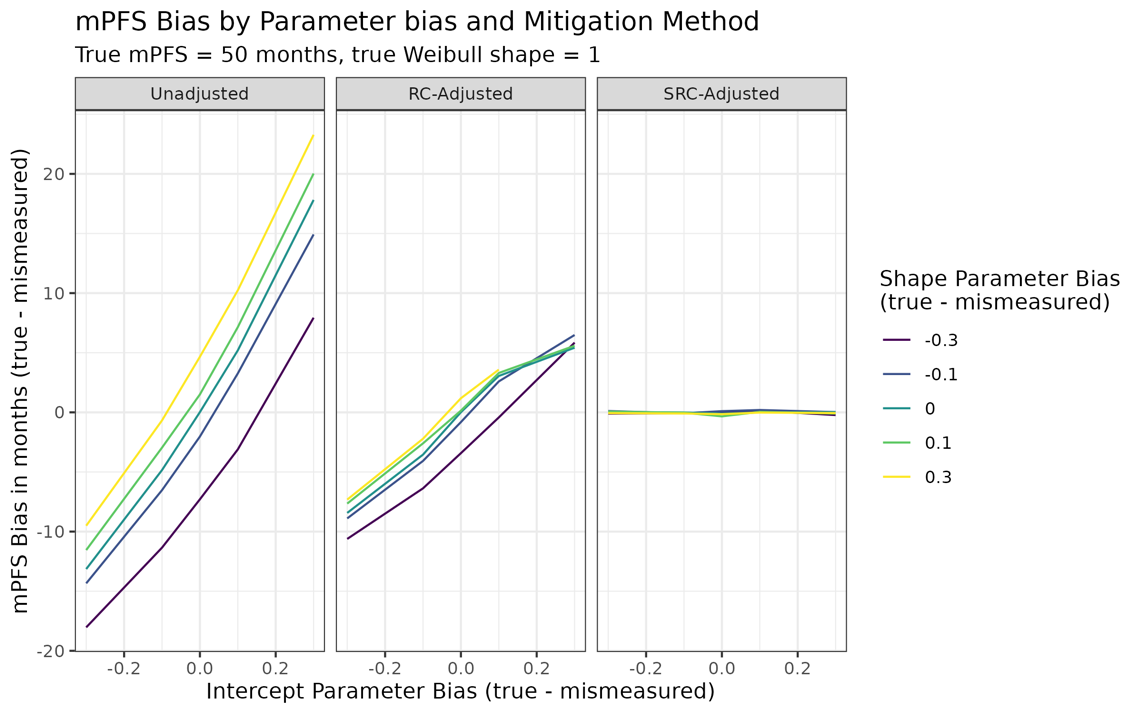


Figure S4: Simulation results when true mPFS = 50 months (Weibull shape = 1). The left panel shows the observed mPFS bias when varying bias in the intercept (by x axis) and bias in the shape (by color). The middle panel shows bias after applying standard regression calibration methods. The right panel shows bias after applying the proposed survival regression calibration method.

#### 95% Confidence Interval Coverage

We estimated 95% confidence interval coverage for mPFS by method using the bootstrap method. Coverage was defined as the proportion of simulated datasets where the 95% confidence interval contained the true mPFS (i.e. under no measurement error). To assess sensitivity to validation sample size, coverage was estimated when sampling either 40% of the study for an internal validation study (Figure S5), or 90% of the study for an internal validation study (Figure S6). The unadjusted mPFS (under measurement error) generally had poor coverage under varying degrees of measurement error. While RC improved coverage in many cases, it still performed poorly under large amounts of bias in the shape and/or scale parameters. SRC yielded coverage of nearly 100% when sampling 40% of the study for internal validation, likely due to the large variability in mPFS estimates. When exploring a larger validation sample, the coverage for SRC remained strong and above 95%.


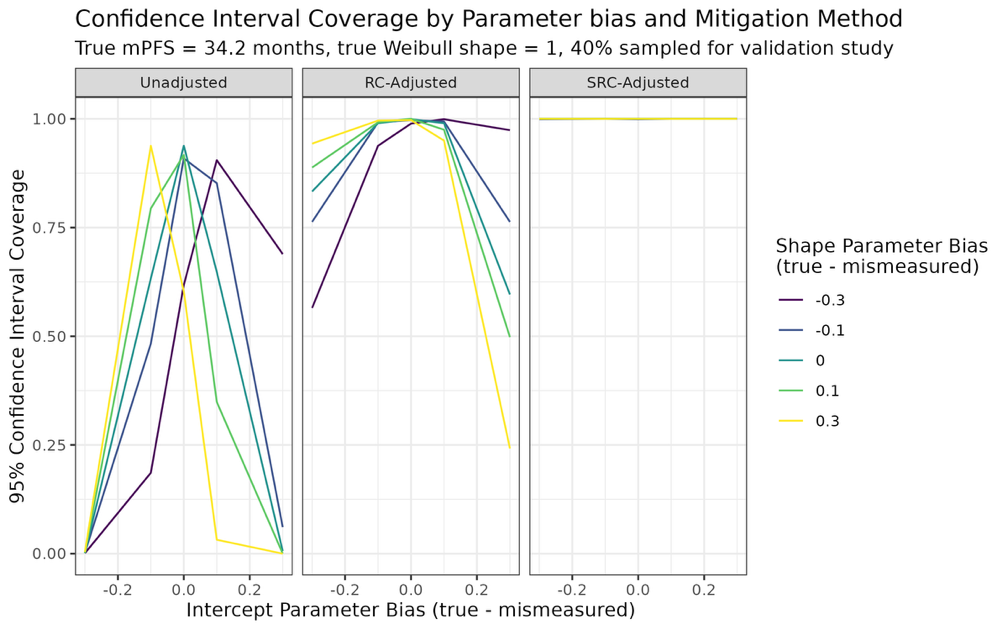


Figure S5: Simulation results of 95% confidence interval coverage when estimating median PFS by method, when 40% of the N=365 study is sampled for an internal validation study. The left panel shows the observed mPFS 95% confidence interval coverage when varying bias in the intercept (by x axis) and bias in the shape (by color). The middle panel shows coverage after applying standard regression calibration methods. The right panel shows coverage after applying the proposed survival regression calibration method.


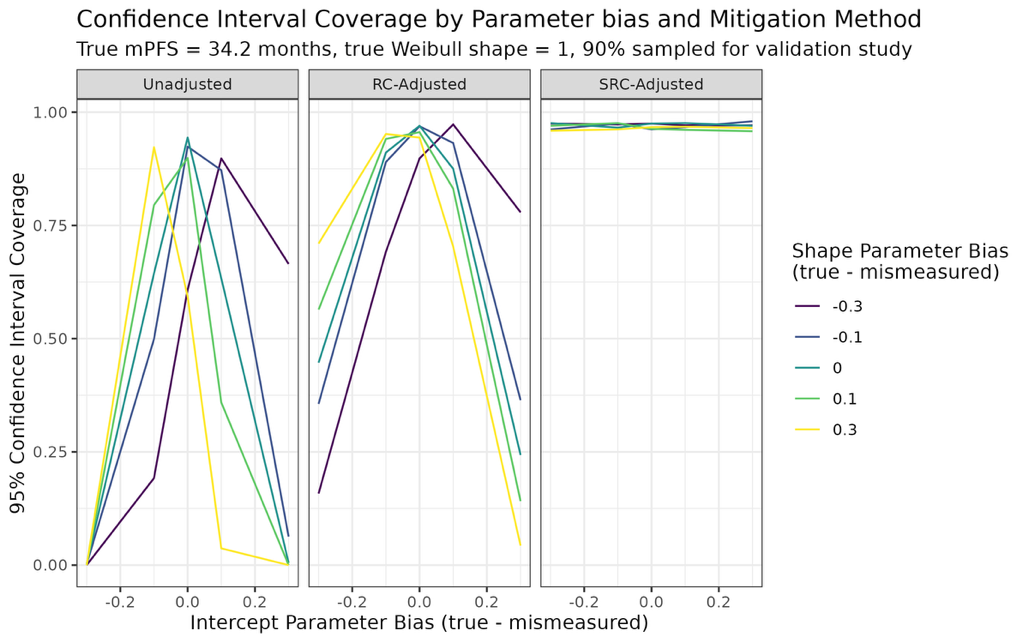


Figure S6: Simulation results of 95% confidence interval coverage when estimating median PFS by method, when 90% of the N=365 study is sampled for an internal validation study. The left panel shows the observed mPFS 95% confidence interval coverage when varying bias in the intercept (by x axis) and bias in the shape (by color). The middle panel shows coverage after applying standard regression calibration methods. The right panel shows coverage after applying the proposed survival regression calibration method.

#### SRC Performance under Model Misspecification

To evaluate performance of SRC under model misspecification, we considered a simulation scenario where data were generated per the Weibull measurement error model detailed in the paper, but a log-logistic model was used to estimate the true and mismeasured parameters and calibrate the outcomes. Methodologically, the process of survival regression calibration is the same: first, an outcome model is fit to the mis-measured time-to-event data in the full study. Then, models are fit to the true and mis-measured outcomes in the validation sample, and the bias between the parameter estimates is calculated. Finally, the mis-measured parameters in the full sample are calibrated by the bias, and the adjusted median time is estimated. While the Weibull model may be a common choice for parametric survival analysis, the log-logistic model may be appealing to researchers in some cases, as the median is equal to its scale parameter. Therefore, when applying SRC using log-logistic models, one could consider estimating bias in the log-logistic scale in order to calibrate estimates of median time to event.

We summarize the simulation results in Figure S7, comparing this mis-measured SRC approach to the other approaches studied in the main text. The results suggest that, while SRC may still yield less bias than other standard RC approaches (and improve estimates of models relying on mis-measured outcomes alone), using a mis-specified model may still yield results that over-estimate median time to event. This underscores the importance of model specification as a topic for further research when implementing regression calibration for time-to-event endpoints.


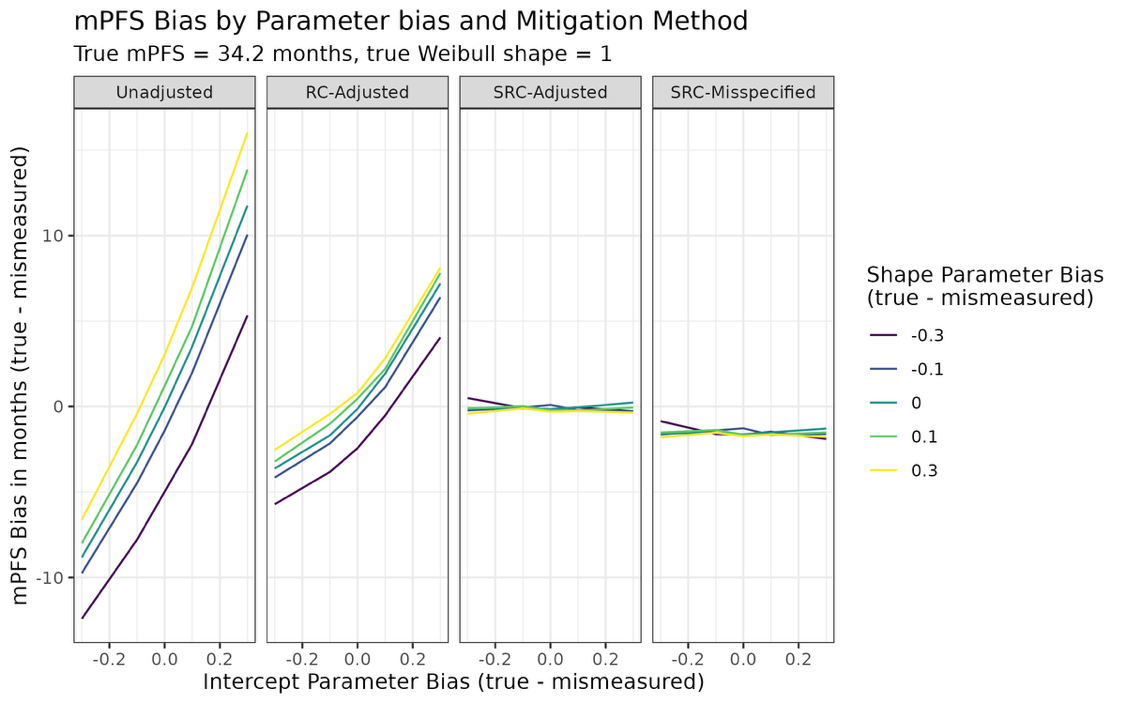


Figure S7: Simulation results when true mPFS = 34.2 months (Weibull shape = 1). The left panel shows the observed mPFS bias when varying bias in the intercept (by x axis) and bias in the shape (by color). The second from the left panel shows bias after applying standard regression calibration methods. The second from the right panel shows bias after applying the proposed survival regression calibration method. The right panel shows bias after applying the proposed survival regression calibration method, but using a misspecified log-logistic model to estimate the bias parameters.

### Code to Implement Survival Regression Calibration

# Load packages ----

library(tidyverse)

library(survival)

# Helper function for calculating Weibull median survival times ----

weib_median_time <- function(linpred, shape, intercept_bias = 0, shape_bias = 0) {

#'@param linpred Numeric: Observed (i.e. mismeasured) intercept parameter in full dataset of interest

#'@param shape Numeric: Observed (i.e. mismeasured) shape parameter in full dataset of interest

#'@param intercept_bias Numeric: estimated bias between true and mismeasured intercept parameter

#'@param shape_bias Numeric: estimated bias between true and mismeasured shape parameter

# Calibrate the intercept and shape parameters

intercept_adj = exp(sum(linpred) + intercept_bias)

shape_adj = shape + shape_bias

# Estimate of median time

res = intercept_adj * log(2) ** (1 / shape_adj)

return(res)

}

# Fit model and estimate parameters using observed data ----

weib_obs <- survreg(Surv(pfs_rwP, pfs_rwP_event) ~ 1, dist = "weibull", data = methods_df)

weib_obs_int <- weib_obs$coefficients[[1]]

weib_obs_shape <- 1/weib_obs$scale

# Fit true and mismeasured models in validation data ----

# True model

weib_true_val <- survreg(Surv(pfs_prog_imwg_confirm, pfs_prog_imwg_confirm_event) ~ 1, dist = "weibull", data = methods_df %>% filter(internal_validation))

weib_true_int_val <- weib_true_val$coefficients[[1]]

weib_true_shape_val <- 1/weib_true_val$scale

# Mismeasured model

weib_obs_val <- survreg(Surv(pfs_rwP, pfs_rwP_event) ~ 1, dist = "weibull", data = methods_df %>% filter(internal_validation))

weib_obs_int_val <- weib_obs_val$coefficients[[1]]

weib_obs_shape_val <- 1/weib_obs_val$scale

# Calculate bias in intercept and shape in validation sample

bias_intercept <- weib_true_int_val - weib_obs_int_val

bias_shape <- weib_true_shape_val - weib_obs_shape_val

# Estimate the observed and calibrated median PFS ----

# Observed mPFS

obs_mpfs <- weib_median_time(

linpred = weib_obs_int,

shape = weib_obs_shape

)

# SRC-calibrated mPFS

adj_mpfs <- weib_median_time(

linpred = weib_obs_int,

shape = weib_obs_shape,

intercept_bias = bias_intercept,

shape_bias = bias_shape

)

# Compare the estimates

obs_mpfs; adj_mpfs
